# Acoustic emission multiplets as early warnings of fatigue failure in metallic materials

**DOI:** 10.1038/s41598-017-13226-1

**Published:** 2017-10-20

**Authors:** S. Deschanel, W. Ben Rhouma, J. Weiss

**Affiliations:** 1 Université de Lyon, MATEIS, UMR 5510, INSA de Lyon, 69621 Villeurbanne, France; 2 ISTerre, CNRS and Université Grenoble-Alpes, CS 40700, 38053 Grenoble cedex 9, France

## Abstract

Fatigue, i.e. the failure of mechanical structures under cycling loading, remains a considerable technological challenge as it occurs unexpectedly when the structure is operating apparently in a safe and steady state regime, without external signs of mechanical deterioration. Here we report for the first time, in different metallic materials, the detection of acoustic emissions specific of fatigue crack growth. These so-called acoustic multiplets are characterized by nearly identical waveforms, signature of a unique source, are repeatedly triggered over many successive loading cycles at the same stress level, and originate from a single location. They mark the slow, incremental propagation of a fatigue crack at each cycle, or the rubbing along its faces. Being specific to fatigue cracking, they can be used as early warnings of crack propagation, which will ultimately lead to structural failure. Their detection and characterization thus open the way towards a new, reliable monitoring of the onset of fatigue cracking during mechanical tests or within structures in service.

## Introduction

Structural components subjected to cyclic loading can fail even if the stress level remains well below the failure stress observed under static or monotonic loading. This mechanical failure mechanism, called fatigue, became a subject of scientific investigation from the beginning of the industrial revolution^1,2^, owing to its importance in naval and rail industries^3^, or more recently in aeronautics^4^, micro-electronics^5^, or nanotechnology^6^. Despite more than 150 years of experimental and theoretical studies, this failure mechanism remains one of the most dangerous for engineering, as it can occur after hundreds to thousands of cycles, without apparent sign of mechanical damage such as a measurable modification of the macroscopic mechanical behavior^7^. It is expected that more than half of mechanical failures in service might be due to fatigue, with obvious, very large economical costs^8,9^.

**Figure 1:**
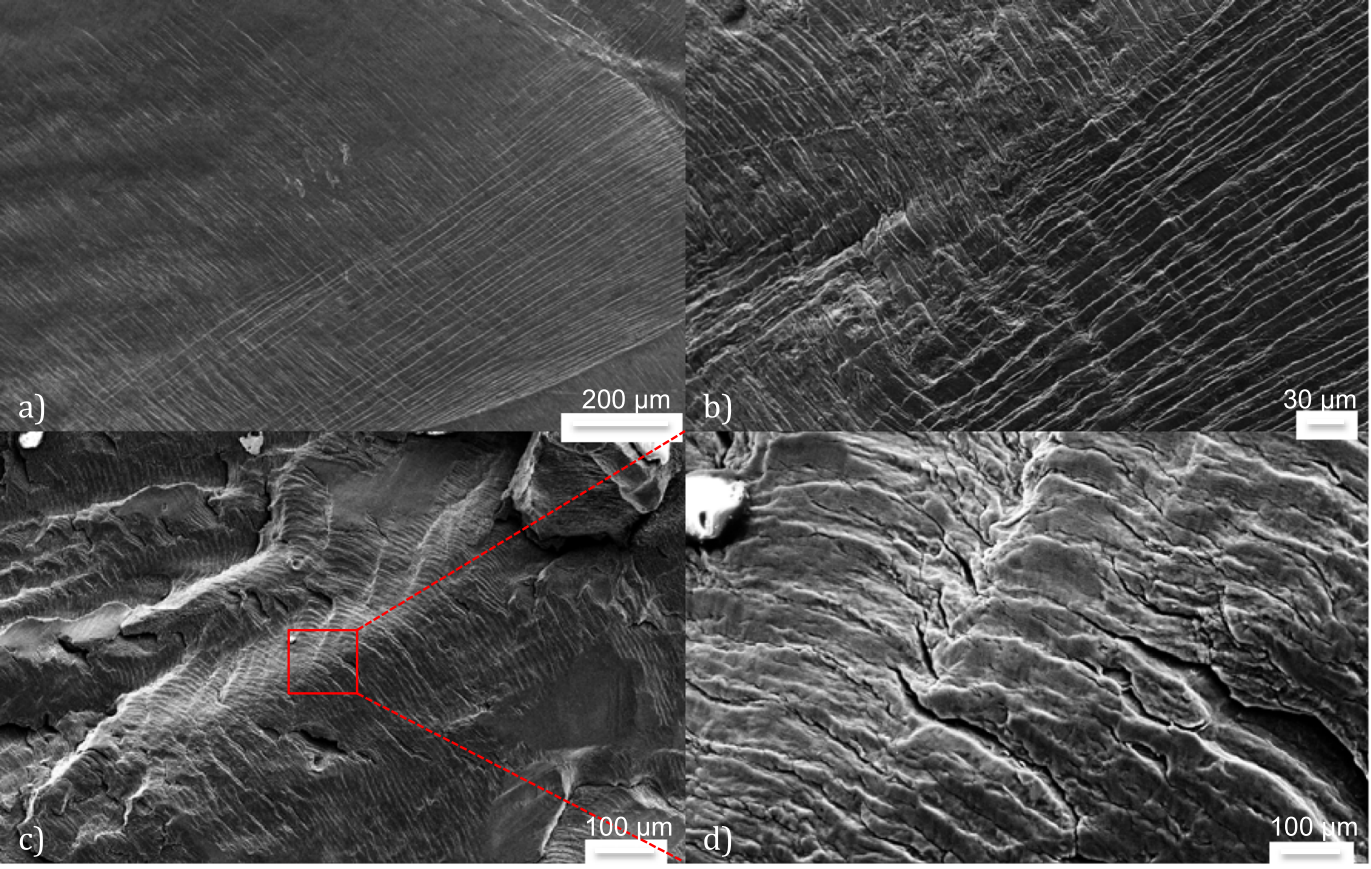
SEM micrographs revealing (**a**) well-defined PSB after 100 cycles, (**b**) signs of crack nucleation after 600 cycles, (**c**,**d**) fatigue striations, unevenness and roughness of the surfaces on a fracture surface after 3000 cycles (fatigue test on pure aluminum at Δε = 0.95%).

In metals, steels and engineering alloys for which the plastic yield stress is often reached during loading or service, the mechanisms of fatigue crack nucleation and propagation, precursors of terminal failure, are well identified. Under cyclic loading, plastic deformation localizes along persistent slip bands (PSB) associated with an irreversibility of slip along different glide planes. As first postulated by Wood^10^, this irreversibility and the associated surface roughness generate stress concentrations, hence sites for crack nucleation^9^. After a stage I of fatigue crack growth, limited to a few grain sizes and during which propagation occurs essentially along primary slip planes, propagation shifts towards a stage II characterized by crack planes perpendicular to the principal tensile stress. During this stage, possibly lasting thousands of cycles, a crack of length *a* advances by small (below few µm) increments $da/dN$ at each cycle, leaving striations on crack surfaces (Fig. 1). Such incremental, repetitive crack propagation is also observed in polymers^11^ or metallic glasses^12^, although the nature of stress concentrators for crack nucleation is different. Unlike generalized damage evenly spread within a structure, the presence of one or few fatigue cracks does not modify significantly the average mechanical properties of the material such as elastic stiffness. As stage II propagation often occurs within the bulk, their detection and monitoring from visual inspection is also problematic, whereas X-ray tomography^13^ can be hardly applied to structural components in service. Propagating slowly without clear signature in terms of macroscopic mechanical behavior, the fatigue crack eventually attains a critical length such that the stress intensity factor at crack tip reaches the fracture toughness during the loading cycle, triggering an unstable propagation and global failure called stage III.

There are many examples of severe, sometimes catastrophic accidents resulting from such undetected fatigue crack propagation ending in structural failure^3,4,14^. If lifetime and safety prediction methodologies based on the non-destructive monitoring of mechanical properties^15^ have been proposed, the early detection and monitoring of fatigue crack growth remains a crucial challenge which motivated numerous studies tempting to detect a modification of material properties, such as electrical resistivity^16^, ultrasonic energy^17^, electromagnetic impedance^18^, or electrochemical properties^19^. A common limitation of these non-destructive methods is that they detect a modification of a physical property averaged at the scale of the structure or the component, hence are hardly sensitive to the effect of a single (or few) crack in its early stages of propagation, and faced with classical signal/noise issues. This can lead to (too) late alarms.

Fatigue crack growth, such as any sudden local change of irreversible strain^20^, is a source of acoustic emission (AE). AE has therefore been proposed as a monitoring tool of fatigue for a long time^21^. Most of these early works as well as more recent ones^22,23^ recorded the global AE activity, such as the number of AE counts, or of detected bursts per cycle. Correlations between these rates and the crack growth rate $da/dN$, when studied, are only significant close to final failure^22^. This illustrates a limitation shared with the other non-destructive methods listed above: tracking the slow crack growth from a global measure is difficult and highly sensible to the signal/noise ratio. This is reinforced by the *non-specific* nature of these AE measurements. Indeed, besides crack growth, different mechanisms can generate AE during fatigue, such as dislocation avalanches^24^, phase (e.g. martensitic) transformations^25^, twinning^25^, or damage^26^, not speaking about environmental/mechanical noise. Sophisticated analyses were proposed to discriminate the AE signatures of different source mechanisms: Shaira *et al*.^25^ used e.g. the k-means approach for clustering AE signals into different classes (representing mechanisms) in 304L stainless steels. Several other approaches in AE pattern recognition and signal source mechanism identification were developed on other materials^27–30^ but are hardly feasible in-service.

Here, we report the discovery of AE bursts *specific* to the existence of fatigue cracks during cyclic loading in different metals and alloys, in the form of nearly identical waveforms repeating at each cycle at nearly the same stress level. Owing to this specificity, they can be easily discriminated from other AE sources or environmental noise, i.e. are less subjected to noise/signal problems, and can be detected much before any modification of macroscopic properties.

## Experiments

We performed uniaxial strain-controlled tension-compression $({R}_{\varepsilon }=\frac{{\varepsilon }_{min}}{{\varepsilon }_{max}}=-1;\,{\rm{\Delta }}\varepsilon ={\varepsilon }_{max}-{\varepsilon }_{min}=cst)$ as well as stress-controlled $({R}_{\sigma }=\frac{{\sigma }_{min}}{{\sigma }_{max}}=-1;\,{\rm{\Delta }}\sigma ={\sigma }_{max}-{\sigma }_{min}=cst)$ cyclic fatigue tests on several face-centered cubic (FCC) metals under a loading frequency of 0.1 Hz or 1 Hz, using two different hydraulic machines (see Table 1).

**Table 1:** Summary of testing conditions and AE setups.

Material	Test conditions								AE					Cycles to failure (N)
	Sample dimensions	Machine	Type of test	Frequ ency	Strain or stress ratio	Amplitude applied	Strain-rate (s^−1^) σ–*rate (MPa.s* ^−1^ *)*	Nb of tests	AE sensors (PreAmp)	Threshold (V)	PDT-HDT-HLT (µs)	WF sampling rate	1^st^ multiplet (% lifetime)	
Aluminum	Gauge lentgh l:18 mm Ø:9 mm	Mateis hydraulic machine	Strain imposed	0.1 Hz	Rε = −1	Δε = 0.5%	1 × 10^−3^	2	Nano30 (PAC) (PreAmp: 60 dB)	0.06	300–600–1000 (or100)	5 MHz	24–59%	11600–15500
						Δε = 0.95%	1.9 × 10^−3^	8		0.05–0.06		1,2,5 MHz	40–70%	3000–3800
						Δε = 1.5%	3 × 10^−3^	1		0.05		5 MHz	—	580
			Stress imposed		Rσ = −1	Δσ = 50MPa	10	1		0.05		2 MHz	59%	17200
						Δσ = 62MPa	12.4	1		0.05			45%	5300
	l:32 mm Ø:14 mm	MTS	Strain imposed	1 Hz	Rε = −1	Δε = 0.76%	1.5 × 10^−2^	2		0.09–0.11		1 MHz	64–82%	1360–5040
						Δε = 1.5%	3 × 10^−2^	1		0.09			56%	1450
304L	l:18 mm Ø:4 mm	Mateis hydraulic machine	Strain imposed	0.1 Hz	Rε = −1	Δε = 0.95%	1.9 × 10^−3^	2	µ80 (PAC) (PreAmp: 40 dB)	0.06	300–600–1000	5 MHz	75%	320–420
Copper Alloy	l:18 mm Ø:4 mm					Δε = 0.87%	1.7 × 10^−3^	1		0.06			8%	470
Pure Copper	l:18 mm Ø:4 mm					Δε = 0.37%	7 × 10^−4^	1		0.06			65%	3080
						Δε = 0.37%	7 × 10^−4^	1	Nano30 (PreAmp: 40 dB)	0.06			93%	4140

**Figure 2:**
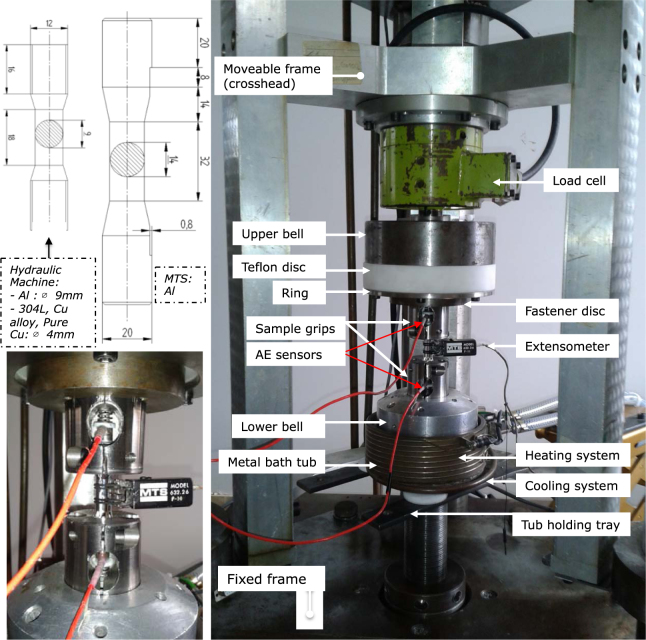
Setup for cyclic fatigue tests (MATEIS hydraulic machine) with AE acquisition and dimensions of the cylindrical specimens for the MATEIS hydraulic machine and the MTS. *Photo courtesy of MTS Systems Corporation*.

The results presented below were obtained on a 99.95% pure aluminum with a polycrystalline structure consisting of large elongated grains, ~10 mm for the major axis and ~3 mm for the minor axis. We chose to study preferentially pure aluminum in order to restrict the potential sources of Acoustic Emission (AE) to collective dislocation motions and microfracturing. Indeed, impurities and phase transformations in a material are possible AE sources^25^ but do not exist for pure aluminum. Other materials were also investigated, including a 304L austenitic stainless steel, which polycrystalline samples were subjected to a homogenization heat treatment at 1050 °C for 1 h and subsequent water quenching. The mean grain size was 50 μm. Polycrystalline electrolytic copper (Cu-a1 -Cu-ETP-) with at least 99.9% Cu, as well as a Copper-Cobalt-Beryllium alloy were also studied. The results obtained with these materials are presented in the Supplementary Information (SI). Two AE piezoelectric transducers were placed on the heads of the cylindrical specimen (58 mm apart) for the tests performed on the MATEIS hydraulic machine (Fig. 2) and on flat sections of the specimens for MTS tests. AE bursts were detected above a voltage threshold and their waveforms (WF) saved (see Methods).

## Results and Discussion

**Figure 3:**
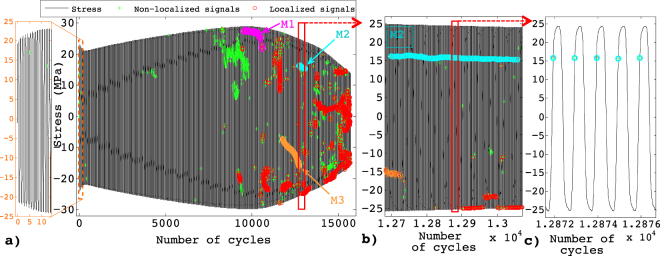
(**a**) Acoustic activity during a fatigue test at Δε = 0.5% on aluminum at 0.1Hz: stress vs number of cycles with non localized AE signals (green crosses) and localized signals (red circles). Magenta, cyan and orange clusters correspond to typical examples of multiplets: respectively named M1, M2 and M3 and analyzed in details in Figs 3, 4 and 6. (**b**) Enlargement on M2 and (**c**) on some loading cycles.

**Figure 4:**
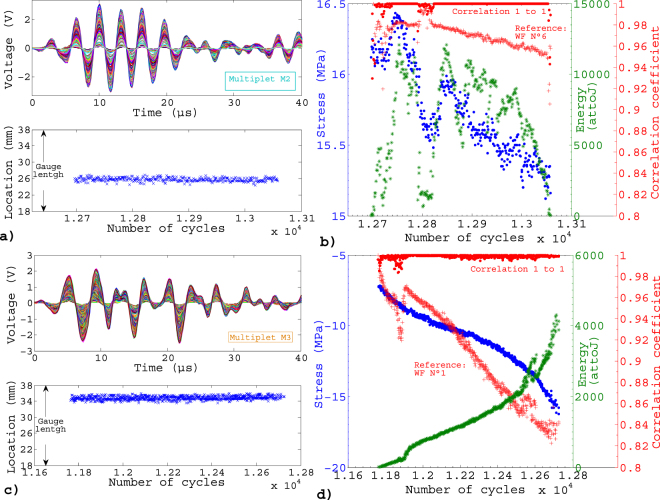
(**a**) Waveforms (WF) composing multiplet M2 from Fig. 2 and location of the localized signals (AE signals occurring every cycle at ${\rm{\sigma }}\approx 16$ MPa during ~360 cycles). (**b**) Corresponding triggering stress (blue) and energy (green) of the WF over the cycles, as well as correlation coefficients of one WF of M2 as a function of the following one in the multiplet (closed red circles) and correlation coefficients between the 6^th^ WF and all the others (red crosses). (**c**) WF composing multiplet M3 from Fig. 2 and location of the events (AE signals occurring every cycle during more than 900 cycles). (**d**) Same as (**b**) for multiplet M3, with the 1^st^ WF taken as reference for the correlation coefficients (red crosses).

**Figure 5:**
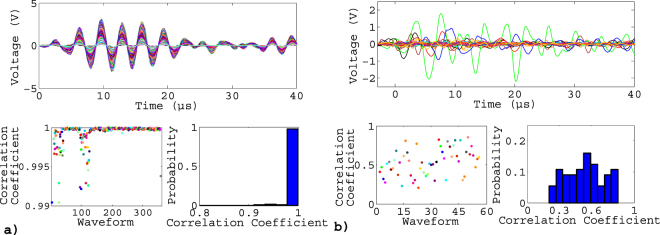
Case of a fatigue test at Δε = 0.5% on aluminum at 0.1Hz. (**a**) Waveforms (WF) composing a multiplet (M2 of Fig. 2); correlation coefficients between one WF and the following one, and corresponding histogram. (**b**) same for WF taken randomly, outside the multiplets, throughout the test. Correlations were calculated over the first 40 µs of the waveforms.

The occurrence of these AE signals during the loading (number of cycles, *N*), at specific level of stress σ within the cycle is demonstrated in Fig. 3, for a test on Aluminum. A remarkable feature in this representation is the presence of clusters consisting of bursts triggered every cycle at almost the same, or at a slowing evolving, stress. These sequences can last hundreds of cycles. Three of those clusters are highlighted in Fig. 3 as typical examples for further analysis. The bursts of a given cluster are characterized by very similar waveforms, with cross-correlation coefficients always larger than 0.8, generally greater than 0.99 (Fig. 4b and d). This clearly differentiates these bursts from isolated ones, whose waveforms show correlations in the range 0.3–0.8 (Fig. 5), this remaining correlation resulting from the resonant character of the AE transducers. These repeating bursts are reminiscent of repeating earthquakes, or *multiplets*, first identified in the 80’s^31^. In seismology, these multiplets are interpreted as repeated stress releases at a same asperity along the fault. In this case, the repeatability is not related to a cyclic loading but to a stick-slip mechanism under a slow far-field driving. We report, in this paper, AE multiplets characterized by highly correlated waveforms (correlation coefficients between WF > 0.8) that are repeatedly triggered over many successive loading cycles at nearby stress levels. AE multiplets have been identified during the monotonic compressive failure of a salt sample at the laboratory scale^32^, but this is the first time that they are reported during fatigue.

Using these cross-correlations, the 1D-localization of the sources of these bursts along the sample, and relatively to other members of the multiplet, can be determined with improved accuracy^32–34^ (see Methods). The results argue for a unique source for each multiplet (Fig. 4a and c), without perceptible evolution of the location during cyclic loading from our measurements.

We interpret these multiplets as the specific signature of stage II incremental fatigue crack growth. Indeed, they are not experimental artifacts, as (i) they are localized inside the samples, (ii) we recorded them during tests performed with two different loading machines (iii) but not during the cyclic loading of samples below the elastic limit, or during the early stages of fatigue. In aluminum or copper alloys, the only possible physical sources of AE are plasticity (dislocation motion), crack nucleation, growth and rubbing of existing crack faces. Dislocation avalanches, which represent the unique source of AE bursts during the first tens of cycles corresponding to the cyclic-hardening stage (Fig. 3a), consist of non-repeating emissions distributed around the macroscopic plastic yield in both tension and compression^24^. If multiplets can be observed either in tension or compression, they are never symmetric, and not triggered at plastic yield. Under conditions for which the plastic zone ahead of crack tip is larger than the grain size, a situation encountered in our tests on ductile materials where macroscopic plastic yield is crossed over at each cycle (Fig. 3c), stage I crack growth is very short^7^, hence hardly compatible with the repeatability of multiplets over hundreds of cycles. Finally, the examination by scanning electron microscopy (SEM), after interrupted tests, of the surface of aluminum samples loaded under ${\rm{\Delta }}\varepsilon =0.95 \%$ revealed well-defined PSB after 100 cycles, and signs of crack nucleation after 600 cycles (Fig. 1), whereas AE multiplets were never detected before 1200 cycles under these conditions (Table 1).

**Figure 6:**
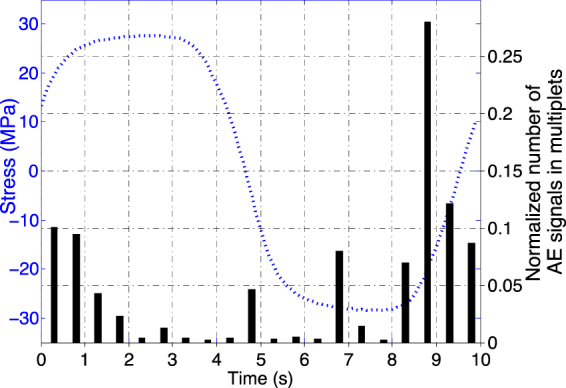
Detection of multiplets within the cycle for fatigue tests at imposed strained (${\rm{\Delta }}{\rm{\varepsilon }}\,=$ 0.5% and 0.95%, ${R}_{\varepsilon }=-1,0.1\,{\rm{Hz}})\,$ on Aluminum. The dotted blue line represents a typical loading cycle for such strain-imposed fatigue tests.

When detected under increasing tension ($\sigma  > 0;\,\dot{\sigma } > 0$), we argue that these repeating bursts are the direct signature of incremental crack growth by an amount *da* at each cycle. Consistently, multiplets were almost never detected under decreasing tension ($\sigma  > 0;\,\dot{\sigma } < 0$) (Fig. 6). Considering a crack of length *a* extending uniformly by an increment *da* along the crack front of length *l*, the AE wave energy scales as ${E}_{AE} \sim {K}_{I}l{(da)}^{3/2}$, where ${K}_{I} \sim \sigma \sqrt{a}$ is the stress intensity factor at crack tip^35^. Although fatigue crack growth is triggered for *K*_*I*_-values much below the fracture toughness $\,{K}_{Ic}$, and the conditions for Linear Elastic Fracture Mechanics are not obeyed in ductile materials, one can reasonably assume incremental growth to be, in average, triggered at lower tensile stresses when the crack length increases. This trend was generally observed, although sometimes accompanied by shorter term fluctuations (see e.g. Fig. 4b and SI).

Although multiplets can be tracked over hundreds of cycles, they generally fade out before final failure (Fig. 3). This does not necessarily mean that the associated crack stopped growing. In these ductile samples, multiple cracks are activated and the final cyclic softening stage is characterized by extensive damage. We have to bear in mind that the waveform $W(f)$ results from the convolution of the source signal $S(f)$ and of transfer functions in the frequency domain depending on the acquisition chain: $W(f)=S(f)\times G(f)\times D(f)$. $G(f)$ describes the effects of wave propagation within the medium (usually studied using a Green’s function approach) and $D(f)$ describes the characteristic response of the detection system (i.e. sensor and detection electronics). Assuming that the medium $G(f)$ changes little over a short interval during the test, two similar AE signals received by one sensor$\,{W}_{1}(f)\approx {W}_{2}(f)$, in a small period of time, imply two similar sources ${S}_{1}(f)\approx {S}_{2}(f)$ as $D(f)$ is unchanged. The initial, most energetic part of the signal (0–40 µs in our analyses), mainly controlled by the source characteristics $S(f)$, remains highly correlated over most of the multiplet lifetime, then decorrelates suddenly (Fig. 4b). This final decorrelation may indicate a previously isolated crack starting interacting and coalescing with others, or, under compression, a modification of the characteristics of the asperity, hence changing significantly the emitted waveforms.

**Figure 7:**
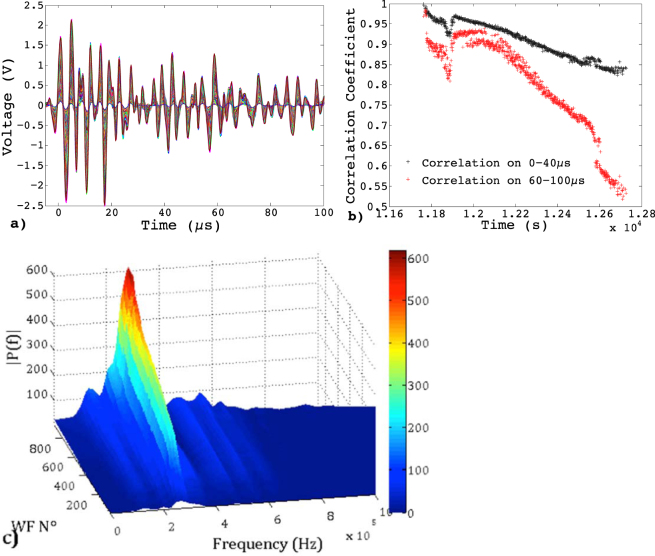
(**a**) 933 waveforms of multiplet M3 of the fatigue test at Δε = 0.5% on aluminum at 0.1 Hz (Fig. 2). (**b**) Correlation coefficients between the first WF and all the others, when the correlation is performed on the first 40 µs (black dots) and on the interval 60–100 µs (red crosses) of the waveforms corresponding to the coda waves. (**c**) Evolution of the power spectral density during multiplet M3.

On the other hand, the limited but progressive decorrelation of the waveform during the multiplet lifetime might result from slowly evolving source characteristics, and/or from a modification of the traversed medium $G(f)$ as the result of increasing damage. This last mechanism is better characterized by the evolution of the diffusive coda (sampled in the interval 60–100 µs in Fig. 7a for multiplet M3 of Fig. 3). This coda, resulting from multiple scattering of the emitted elastic wave on internal defects, samples the damage spread within the material^36^. Its decorrelation rate during the multiplet lifetime is much larger than that of the more energetic part of the waveform (Fig. 7b), signing a progressive damaging during fatigue cycling of this Al sample. This is also emphasized with the evolution of the power spectral density during the multiplet, shown for multiplet M3 in Fig. 7c. Similar results were obtained for the other samples and materials studied. Therefore, using the repetitive AE at each loading cycle as a “natural” generator of a nearly identical source, such analysis of the diffusive coda could be used to track the average evolution of damage.

During the first 60% of fatigue life, multiplets were mainly detected under tension, hence related to crack growth. In the later stages of fatigue, we also observed AE multiplets under decreasing ($\sigma  < 0;\,\dot{\sigma } > 0$) and, to a lesser extent, increasing ($\sigma  < 0;\,\dot{\sigma } < 0$) compression (Fig. 6). The unlocking of a frictional asperity during either crack closure or re-opening, i.e. rubbing of crack faces, is the most plausible source mechanism, leading to local flattening and wearing of the rough crack surfaces clearly visible on post-mortem SEM photographs (Fig. 1c). In this case, we expect the wave energy to scale as ${E}_{AE} \sim |\sigma |Su$ where *u* is the average slip over the surface *S* of the asperity. Considering that the characteristics of the asperity evolve slowly during cycling, a proportionality between ${E}_{AE}$ and $|\sigma |$ is in agreement with our observations (Fig. 4). However, the ratio ${E}_{AE}/|\sigma |$ was generally observed to slowly increase with increasing cycling, which could be interpreted as an increasing asperity surface *S* and/or average slip *u*. If they sign the presence of a fatigue crack, these multiplets under compression reveal however relatively little information about the crack growth process itself.

To summarize, we reveal for the first time the presence of repeating nearly identical acoustic emissions during fatigue testing of metals. Originating from a unique source and triggered at each loading cycle at close stress levels, these AE multiplets are a specific signature of incremental fatigue cracking. Their detection thus open the way towards possible early warnings of fatigue failure during mechanical tests, or within structures in service, as the first multiplets were generally detected before 2/3 of fatigue life, before any macroscopic sign of material damage such as cyclic softening (Table 1).

## Methods

### Mechanical tests

Uniaxial strain- or stress-controlled low cycle fatigue tests were performed at room temperature on cylindrical samples (dimensions are given in Table 1 and drawings are presented in Fig. 2). Two different hydraulic machines were used: a machine designed and realized at MATEIS Lab^37,38^ and a MTS -Mechanical Testing System- machine. The MTS machine is a uniaxial machine with a 50kN load cell and hydraulic cylinders to hold the sample. The MATEIS machine has a loading capacity of 10kN and was specifically designed to generate little noise compared to other hydraulic machines, as relative movements of mechanical parts are avoided during cycling, then minimizing noise from friction. Moreover, in order to reduce the influence of external vibrations, the machine is disconnected from the ground vibrations thanks to air chambers. Finally, the sample is fixed with a specific setup (Fig. 2) allowing an acoustic confinement from the rest of the machine. Strain is measured thanks to an MTS-extensometer. For strain-controlled tests, the strain ratio ${R}_{\varepsilon }=\frac{{\varepsilon }_{min}}{{\varepsilon }_{max}}=-1$ was equal to −1 (tension-compression), the total strain was imposed with a constant amplitude ${\rm{\Delta }}\varepsilon ={\varepsilon }_{max}-{\varepsilon }_{min}$ varying from 0.5% to 1.5% under a loading frequency of 0.1 Hz or 1 Hz. The stress-imposed tests were performed at 0.1 Hz with a stress ratio ${R}_{\sigma }=\frac{{\sigma }_{min}}{{\sigma }_{max}}=-1$. The conditions are summarized in Table 1. Stress and strain were recorded up to the macroscopic failure of the sample.

### Acoustic emission recording

Acoustic emission is continuously monitored during the tests using a PCI2 Mistras data acquisition system of Euro Physical Acoustics S.A. (EPA) with a 40 or 60 dB pre-amplification and a 50 kHz–1.2 MHz bandwidth and a 40 MHz maximum technical sampling rate. However, the hardware clock is set to an accuracy of 0.1 µs which represents the irreducible synchronization accuracy between the clocks of the different transducers. On the other hand, the AE waveforms are sampled at a frequency varying from 1 to 5 MHz, depending on the tests (see Table 1), but this does not alter the clock accuracy. Our measurements are achieved with two resonant piezoelectric sensors (either Nano30 EPA or micro80 EPA with a peak of resonance at 140 kHz and 300 kHz, respectively) coupled to the material with silicon grease. For the tests performed on the MATEIS machine, the sensors are placed on the heads of the specimens and maintained thanks to springs in tailor-made grips (Fig. 2). For the tests performed on the MTS, the sensors are maintained thanks to Teflon tape on the flat sections of the specimens. We do not record the full acoustic signal over the entire test duration, which can last several hours. We are working instead in an automated detection mode. Transient elastic waves are selected from the background noise above a given amplitude threshold ${A}_{th}$ which varied from 0.05 to 0.11V depending on the test (see Table 1). The data acquisition system determines the AE signal arrival times, ***t***_1_ and ***t***_2_, at sensors 1 and 2 respectively, corresponding to the first threshold crossing of the signal. These arrival times are known with a precision of 0.1 µs (see above) with an important consequence in terms of localization accuracy (see below). Acoustic activity is recorded during all the different tests performed on different machines and materials. Several parameters determined from the waveforms are recorded for each acoustic event: maximum amplitude, energy, rise time, counts (number of threshold crossings) and duration. The amplitude distribution covers the range between ${A}_{th}$ and 10 V, and the energy ${E}_{AE}$ is obtained by integration of the squared signal over the duration of the waveform. An ultrasonic transducer is used to generate well-known pulses in order to calibrate the tests, in particular to set up the acquisition parameters for our materials: peak definition time (PDT), hit definition time (HDT), hit lock time (HLT), time constants to individualize the waveforms (values summarized in Table 1). In addition, we measured the mean wave propagation velocity ${\boldsymbol{v}}$ for each sample at room temperature (aluminum: 5000 m/s, 30 4L: 5800 m/s, Cu alloy and Pure Cu: 3570 m/s).

### Correlation coefficients

All the AE waveforms are extracted for analysis. A cross-correlation study is conducted on the waveforms (within the multiplets, or all over the test): a measure of similarity between two signals as a function of a time lag^39^. The cross correlations between two waveforms, *x* and *y*, are systematically calculated from ${R}_{xy}(\tau )=\frac{(x(t)-x)(y(t+\tau )-y)}{{\sigma }_{x}{\sigma }_{y}}$, $\tau \,$is the time delay and ${\sigma }_{x}$the standard deviation of $x$ (the correlation is generally performed here on the first 40 µs of the waveforms). The sequence is thus normalized so the autocorrelations at zero lag are identically 1.0. Then, the time lag $\delta t$ between the two signals maximizing the cross-correlation sets the time where the signals are best aligned. This time lag $\delta t$ will be used for a new method of AE source localization, described below.

The correlation coefficient between the two waveforms is given by the maximum of the normalized sequence. For the representation of the signals (e.g. Fig. 4), the different waveforms in a cluster are shifted and aligned on the maximum of correlation.

### Localization procedure

AE signals detected by the two sensors can be located along the specimen length when the difference of arrival time between sensor 1 and sensor 2, ${\rm{\Delta }}t$, is smaller than ***d***/***v*** (**d** being the distance between the two sensors and ${\boldsymbol{v}}$ the wave velocity in the material).

#### Classical procedure

In AE, localization is classically performed from triangulation based on the arrival times determined from first threshold crossings. In our case, with two sensors, only a 1D-localization along the specimen length is possible from ${\rm{\Delta }}t={t}_{1}-{t}_{2}$. The position of the source is given by $p={\rm{\Delta }}t\frac{v}{2}+\frac{d}{2}$. The limitations of this method are that ${\rm{\Delta }}t$ strongly depends on the chosen threshold and that the first threshold crossing may not be representative of the arrival time if the first rise is not steep enough.

**Figure 8:**
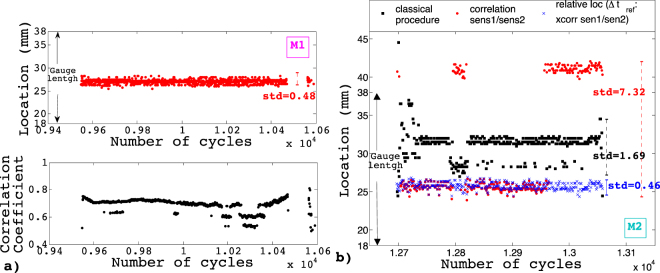
Fatigue test at Δε = 0.5% on aluminum at 0.1Hz. (**a**) Localization of the AE signals of multiplet M1 of Fig. 2, using cross correlations of the signals received by the two sensors (Grabec’s methodology) and corresponding correlation coefficients. (**b**) Localization of the AE signals of multiplet M2 of Fig. 2, using the classical procedure (black squares), the Grabec’s methodology (red circles) and the correlation of waveforms within a multiplet (with the reference determined with cross-correlation of the two sensors, blue crosses).

Let’s note ${t}_{1,i}$ and ${t}_{2,i}$ the arrival times of the different waveforms of a given multiplet, for sensor 1 and 2 respectively, given by the first threshold crossing. Using the classical localization procedure, the positions of the different members of the multiplet would be given by ${p}_{i}=(\,{t}_{1,i}-{lt}_{2,i})\frac{v}{2}+\frac{d}{2}$. This methodology gives systematically dubious shifts in the absolute position of the source within the multiplet (Fig. 8b) that would be in contradiction with a common origin.

#### Localization using correlation between waveforms

To improve the accuracy on ${\rm{\Delta }}t$ and to circumvent the limitations mentioned above, Grabec^40^ proposed to cross-correlate the signals of a same event received by different transducers, with ${\rm{\Delta }}t$ maximizing this cross-correlation. However, this methodology is only applicable if the characteristic responses of the two sensors are very similar (i.e. ${D}_{sen1}(f)\approx {D}_{sen2}(f)$) and if the medium crossed by the elastic waves is homogeneous, ensuring ${G}_{sen1}(f)\approx {G}_{sen2}(f)$. Both conditions are hardly fulfilled, especially in a damaged medium. We checked this methodology, and found that in few cases, for AE multiplets recorded during the early stages of damaging, i.e. when the material remains relatively homogeneous, the correlation coefficients between waveforms of a same event recorded at the two sensors over the interval 0–40 µs were in the range 0.5 to 0.8 (Fig. 8a), i.e. slightly larger than coefficients obtained between waveforms randomly selected (Fig. 5b). Then, for waveform *i* within such multiplet, the cross-correlation gives a time lag $\delta {t}_{sen1/sen2}^{i}$ that is used to correct ${\rm{\Delta }}{t}_{i}=({t}_{1,i}-{t}_{2,i})+\delta {t}_{sen1/sen2}^{i}$ and consequently the associated location. In these cases, AE events belonging to a same multiplet are localized around a mean position, with a standard deviation of the order of 500 µm, without spurious shifts (Fig. 8a). However, in most cases, the cross-correlations between waveforms recorded at sensors 1 and 2 are non-significant, i.e. of the order of correlations found for randomly selected waveforms. In those cases, the Grabec’s methodology leads to unphysical shifts in position within a given multiplet (Fig. 8b, red dots), illustrating the shortcomings of this method when the medium is too damaged and therefore the condition ${G}_{sen1}(f)\approx {G}_{sen2}(f)$ no longer fulfilled.

Consequently, we are here taking advantage of the correlated waveforms within the multiplets, recorded at the *same sensor*, to improve the location of the AE signals and to present a new way to map the sources. In this case, $D(f)$ is unchanged by definition, whereas $G(f)$ remains almost unchanged as long as we consider only the initial impulsive part (0–40 µs) of the waveforms (see section 3 above). Inspired by studies of seismic multiplets (or repeating earthquakes) (e.g. refs^33,34,41^), we can perform a *relative* localization of the different waveforms in the multiplet using the time delay $\delta t$ found between two waveforms in a multiplet.

We choose an event as a reference in the multiplet, for which the difference of arrival times between sensor 1 and 2 sets the reference position. This can be done using the first threshold crossing i.e. ${\rm{\Delta }}{t}_{ref}=({t}_{f1,ref}-{t}_{2,ref})$, or alternatively from the Grabec’s cross-correlation methodology mentioned above. These two estimations of the absolute position of the reference are significantly different, generally by more than few mm. This illustrates the intrinsic uncertainty in absolute positioning of AE sources. For each sensor, we then compute the cross correlations of all the waveforms in a multiplet compared with the reference: time delays between each waveform and the reference, $\delta {t}_{1,i}$ and $\delta {t}_{2,i}$, are obtained. A new time of arrival can thus be determined for each wave in the multiplet: ${t}_{1,i}^{corr}={t}_{1,i}+\delta {ft}_{1,i}$ and ${t}_{2,i}^{corr}={t}_{2,i}+\delta {ft}_{2,i}$, giving a new time interval ${t}_{1,i}^{corr}-{t}_{2,i}^{corr}$. Finally, ${\rm{\Delta }}{t}_{i}^{corr}=({t}_{1,i}^{corr}-{t}_{2,i}^{corr})+\delta {t}_{sen1/sen2}^{ref}$ allows to determine the position of the event *i relatively* to the reference: ${p}_{i}^{\ast }={\rm{\Delta }}{t}_{i}^{corr}\frac{v}{2}+\frac{d}{2}$. The irreducible limitation of this method is directly linked to the limitation of the system in terms of clock synchronization between the different sensors (0.1 µs accuracy, see above). For velocities $3500 < v < 5000\,m.{s}^{-1}$, this translates into an uncertainty on the positions between 350 and 500 µm.

We show in Fig. 8b that taking advantage of the correlated waveforms within a multiplet allows suppressing the spurious shifts in position obtained with the classical or the Grabec’s procedures. Moreover, the standard deviation of the position (relatively to the reference) falls down to 460 µm, i.e. very close to the theoretical uncertainty of 500 µm for Aluminum. This strongly argues for a unique source. On the other hand, the *absolute* position of this source remains uncertain to a larger extent (few mm), see above.

### Data availability

The datasets generated during and/or analyzed during the current study are either included in this article (and the Supplementary Information), and/or available from the corresponding author on reasonable request.

## Electronic Supplementary Information

Supplementary Information
