# Supplementary material for: Acoustic emission multiplets as early warnings of fatigue failure in metallic materials
Source: Sci Rep. 2017 Oct 20;7:13680. doi: 10.1038/s41598-017-13226-1 (PMC5654477; doi:10.1038/s41598-017-13226-1)
Supplement: Supplementary file 1 — Supplementary Information [file 41598_2017_13226_MOESM1_ESM.pdf]

Supplementary Information (SI) for

Acoustic emission multiplets as early warnings of fatigue failure in metallic materials

S. Deschanel[1*], W. Ben Rhouma[1] and J. Weiss[2]

[1] Université de Lyon, MATEIS, UMR 5510, INSA de Lyon, 69621, Villeurbanne, France

[2] ISTerre, CNRS and Université Grenoble-Alpes, CS 40700, 38053 Grenoble cedex 9, France

*Correspondence to: stephanie.deschanel@insa-lyon.fr

Supplementary Text: Other materials and/or testing conditions

In the main text, we described multiplets and their characteristics in Aluminum. We show below that similar AE multiplets are also observed on different materials (304L, Cu, Cu-alloy) and in other mechanical testing conditions (different controls: stress controlled tests, at different strain or stress ratio…) and on other loading machines (MTS).

304L stainless steel

Here, we present a fatigue test at imposed strain ($\boldsymbol{\Delta\varepsilon = 0.95\%}$, $\boldsymbol{R_\varepsilon = -1}$) on a 304L stainless steel, performed at 0.1Hz on the MATEIS hydraulic machine.

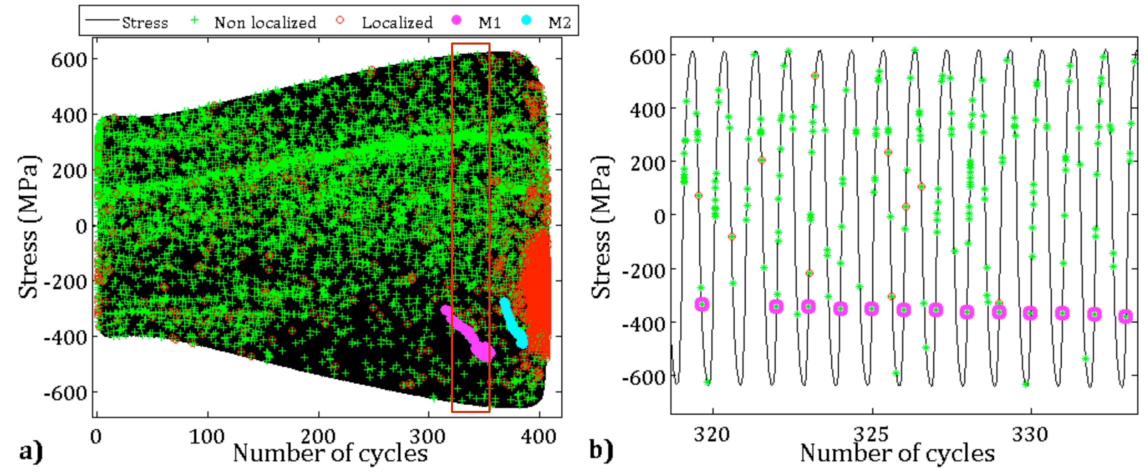

Fig. S1. a) Acoustic activity during a fatigue test at $\Delta\varepsilon$=0.95% on 304L at 0.1Hz: stress vs number of cycles with non-localized AE signals (green crosses) and localized signals (red circles). Magenta and cyan circles correspond to two groups of multiplets. b) Enlargement on some loading cycles around Multiplet M1.

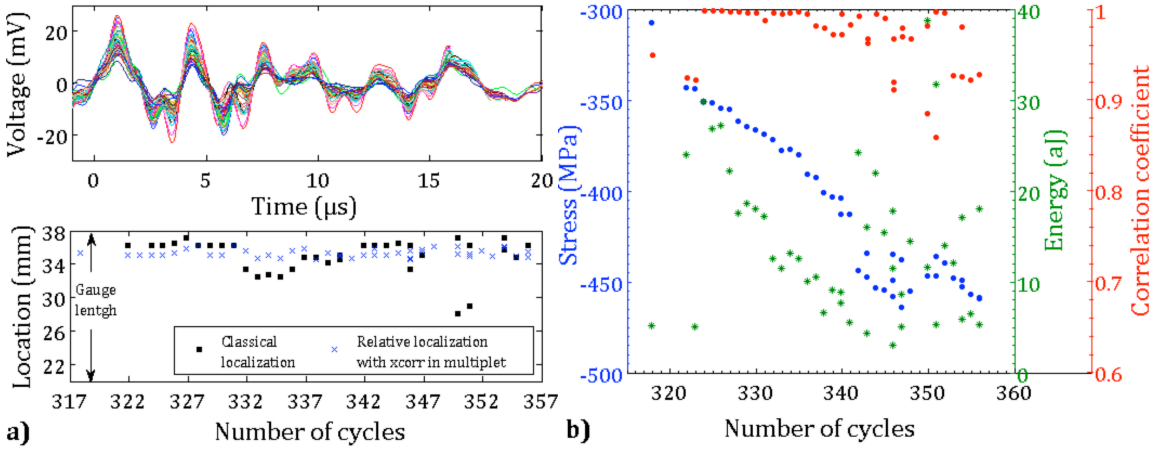

Fig. S2. a) Top subplot: Waveforms (WF) composing a multiplet of the test presented in **Fig. S** (multiplet M1, AE signals occurring every cycle at ~-400 MPa during ~ 50 cycles). Bottom subplot: location of the signals found with the classical method and the new one we present. b) Corresponding stress and energy of the WF over the cycles and in red, the correlation coefficients of one WF of M1 as a function of the following one.

Copper-Cobalt-Beryllium alloy

Similar observations are made on a fatigue test at imposed strain ($\boldsymbol{\Delta\varepsilon = 0.87}$%, $\boldsymbol{R_\varepsilon = -1}$) on a Copper-Cobalt-Beryllium alloy, performed at 0.1Hz on the MATEIS machine.

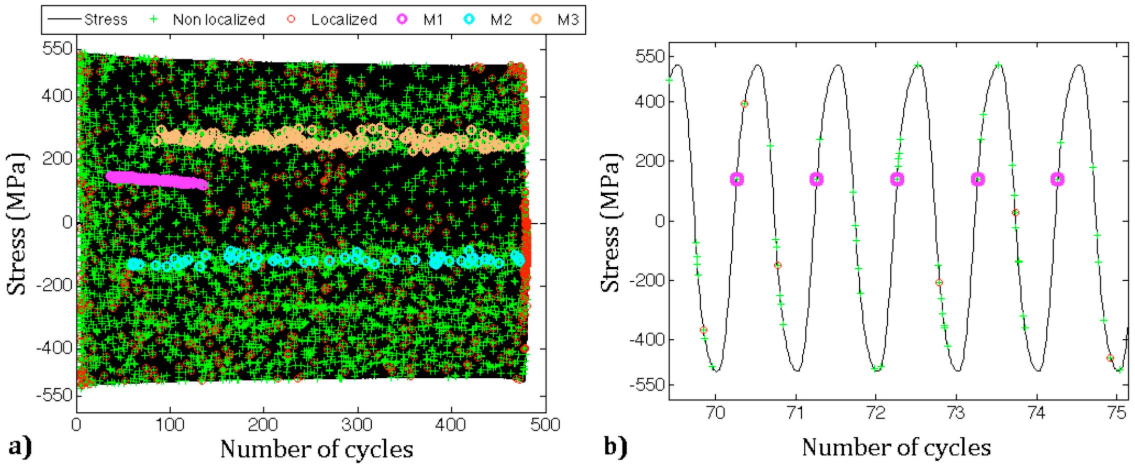

Fig. S3. Acoustic activity during a fatigue test at Δε=0.87% on a Copper-Cobalt-Beryllium alloy at 0.1Hz: stress vs number of cycles with non-localized AE signals (green crosses) and localized signals (red circles). Colored circles correspond to several groups of multiplets. b) Enlargement on some loading cycles around Multiplet M1.of cycles with non-localized AE signals (green crosses) and localized signals (red circles). Colored circles correspond to several groups of multiplets. b) Enlargement on some loading cycles around Multiplet M1.

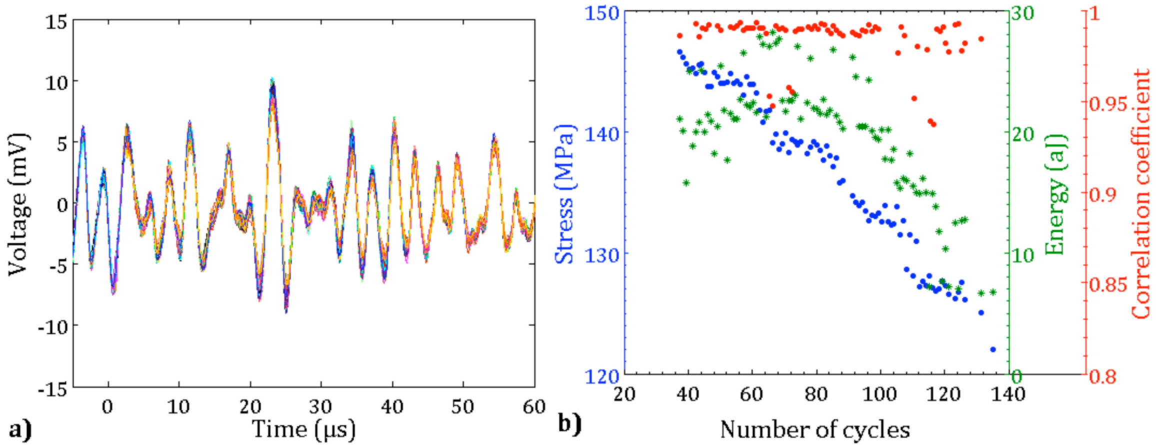

Fig. S4. a) WF composing multiplet M1 of Fig. **S** (AE signals occurring every cycle at ~140 MPa during ~ 100 cycles). b) Corresponding stress and energy of the WF over the cycles and in red, the correlation coefficients of one WF of the multiplet as a function of the following one.

Electrolytic 99.9% Copper

This section presents a fatigue test at imposed strain ($\boldsymbol{\Delta\varepsilon} = \mathbf{0.37}\%$, $\mathbf{R}_{\boldsymbol{\varepsilon}} = \mathbf{-1}$) on a pure Copper specimen at 0.1Hz, performed on the MATEIS machine.

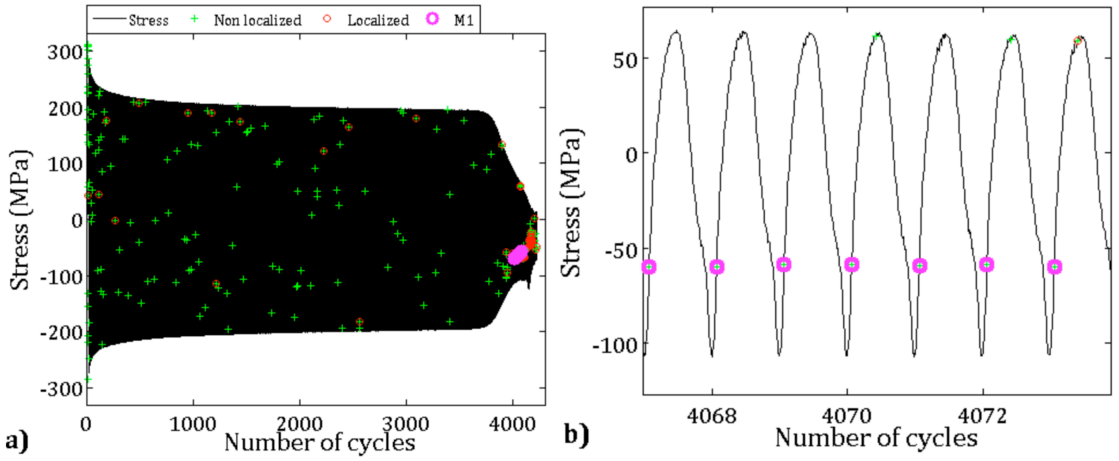

Fig. S5. a) Acoustic activity during a fatigue test at Δε=0.37% on pure Cu at 0.1Hz: stress vs number of cycles with non-localized AE signals (green crosses) and localized signals (red circles). Magenta circles correspond to a group of multiplets b) Enlargement on some loading cycles at the end of the test.

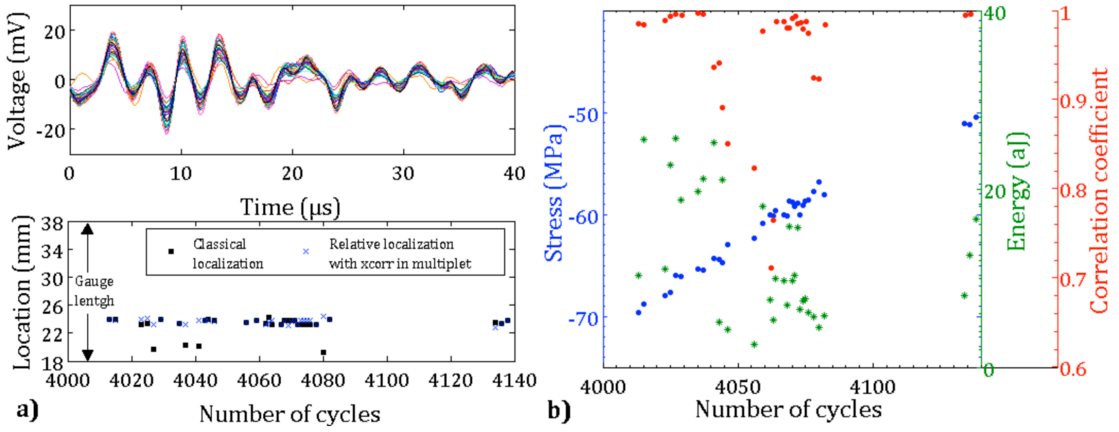

Fig. S6. a) Top subplot: WF composing multiplet M1 of the test presented in **Fig. S** (~ 50 AE signals occurring around [-70, -50] MPa during ~ 150 cycles). Bottom subplot: location of the signals found with the classical method and the relative localization. b) Corresponding stress and energy of the WF over the cycles and in red, the correlation coefficients of one WF of the multiplet as a function of the following one.

99.95% Aluminum: tests at imposed stress

This section presents a fatigue test at imposed stress ($\boldsymbol{\Delta\sigma = 50\text{MPa}\ R_\sigma = -1}$) on a pure Aluminum specimen, performed at 0.1Hz on the MATEIS machine.

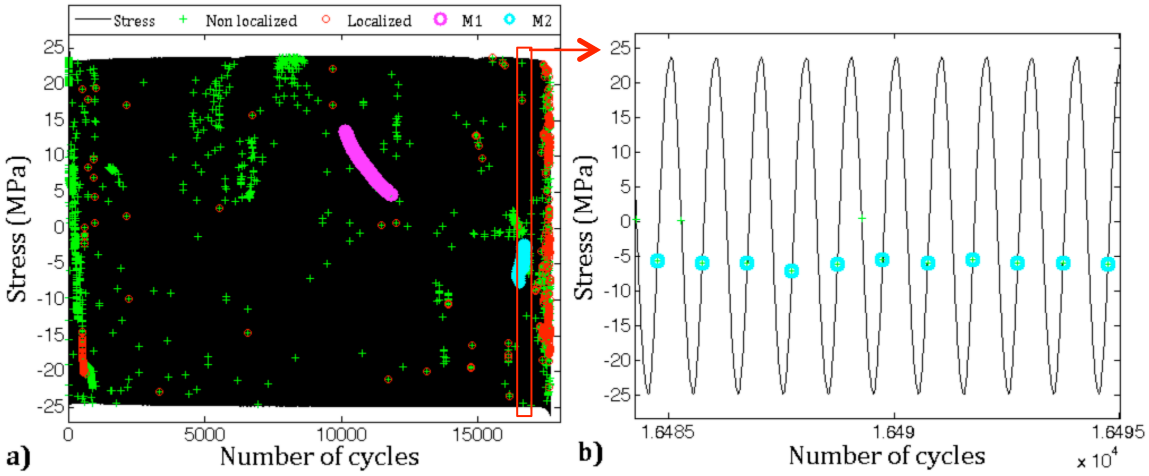

Fig. S7. a) Acoustic activity during a fatigue test at imposed stress (Δσ=50 MPa) on pure Al at 0.1Hz: stress vs number of cycles with non-localized AE signals (green crosses) and localized signals (red circles).. Magenta and cyan circles correspond to two groups of multiplets. b) Enlargement on some loading cycles around Multiplet M2.

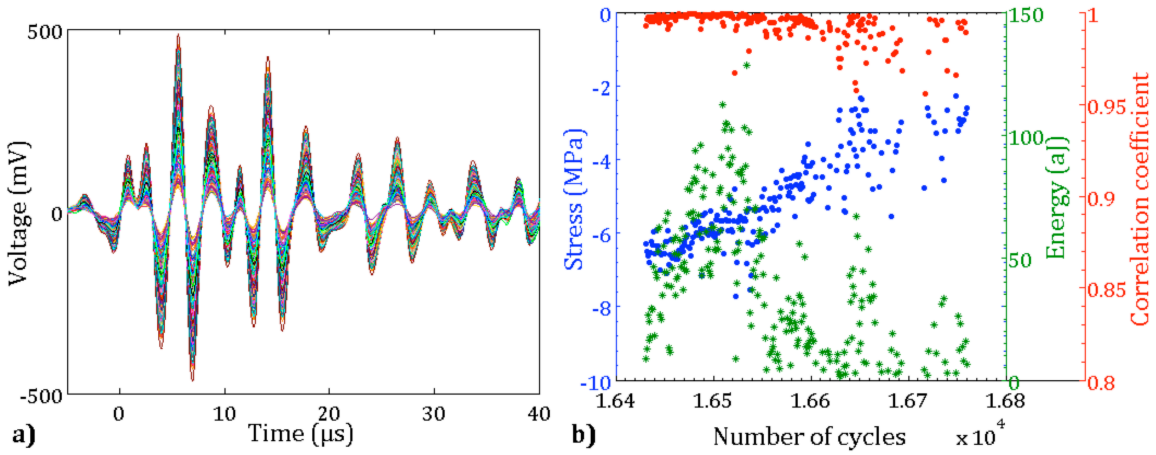

Fig. S8. a) WF composing multiplet M2 (cyan circles on Fig. **S**; AE signals occurring almost every cycle at ~-6 MPa during ~ 300 cycles). b) Corresponding stress and energy of the WF over the cycles and in red, the correlation coefficients of one WF as a function of the following one.

99.95% Aluminum: tests on MTS machine

Finally, we present a fatigue test at imposed strain ($\Delta\varepsilon = 0.75\%$, $R_\varepsilon = -1$) on a pure Aluminum specimen, performed at 1Hz on an MTS machine.

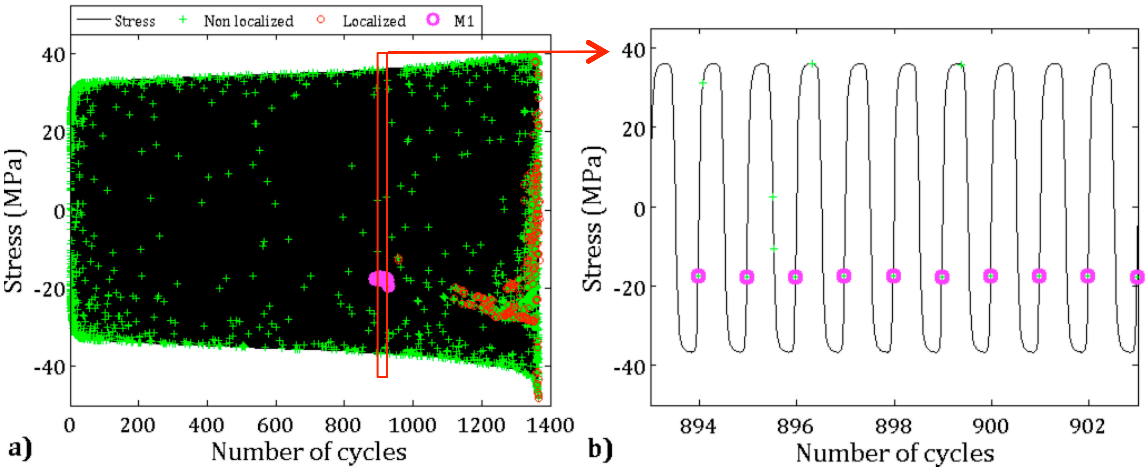

Fig. S9. Acoustic activity during a fatigue test at Δε=0.75% on pure Aluminum at 1Hz on an MTS machine: stress vs number of cycles with non-localized AE signals (green crosses) and localized signals (red circles). Magenta circles correspond to one group of multiplets. b) Enlargement on some loading cycles.

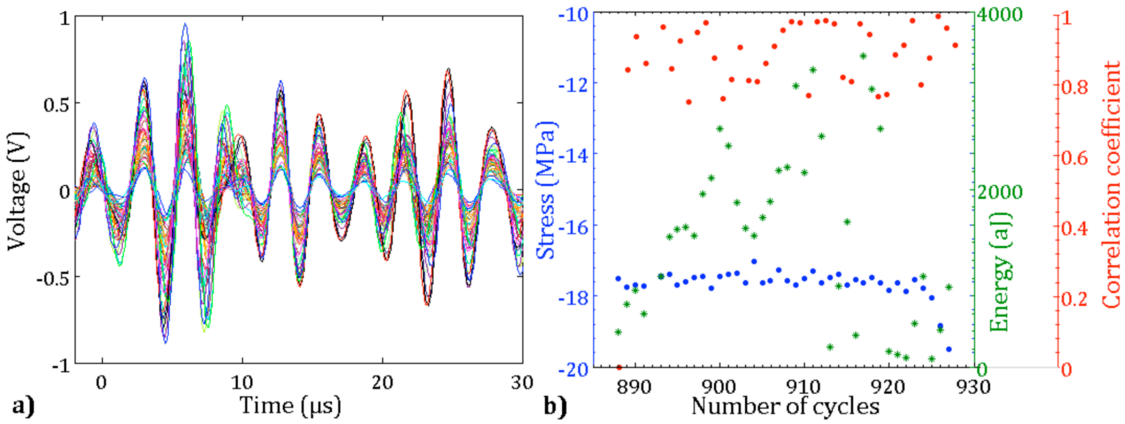

Fig. S10. a) WF composing multiplet M1 of the test presented in Fig. **S** (AE signals occurring almost every cycle at ~-18 MPa during ~ 40 cycles). b) Corresponding stress and energy of the WF over the cycles and in red, the correlation coefficients of one WF as a function of the following one.
